# Lymphoma relapse 1 year or later after immunochemotherapy in DLBCL patients: clinical features and outcome

**DOI:** 10.1007/s10238-024-01306-2

**Published:** 2024-03-01

**Authors:** Hongyun Chen, Jinrong Zhao, Danqing Zhao, Wei Wang, Chong Wei, Zi Wang, Daobin Zhou, Wei Zhang

**Affiliations:** Department of Hematology, Peking Union Medical College Hospital, Chinese Academy of Medical Sciences & Peking Union Medical College, Beijing, 100730 China

**Keywords:** Diffuse large B-cell lymphoma, Relapse, Risk factors, Survival

## Abstract

Despite great advances in treatment, 30–40% of patients with DLBCL undergo relapses. Patients with a relapse within 1 year or beyond have a distinct outcome. Few clinical characteristics and survival data in the Chinese population have been published. We aimed to define the incidence and clinical features of DLBCL patients with very early relapse after front-line immunochemotherapy who may benefit greatly from the emerging chimeric antigen receptor T-cell therapy. Data of 564 DLBCL patients were analyzed. Among the 413 patients achieving a first complete remission, 59 underwent relapses: 32 patients (54.2%) relapsed within 1 year, and 27 patients (46.8%) relapsed 1 year or more. Patients relapsing within 1 year, in comparison with the other group, showed an inferior risk profile at diagnosis: elevated lactate dehydrogenase level (*P* = 0.002), high Eastern Cooperative Oncology Group performance score (*P* = 0.02), and high international prognosis index (*P* = 0.004). As expected, a worse overall survival was observed in the early relapse group. Multivariate analysis for OS showed that relapse within 1 year was an independent parameter for reduced overall survival (HR 0.241, *P* = 0.002).

## Introduction

Diffuse large B cell lymphoma (DLBCL) represents the most common histologic subtype of aggressive non-Hodgkin’s lymphoma (NHL), comprising around 40% of all malignant lymphoid neoplasms [1]. Two dramatic advances have led to great improvements in the outcome and survival of patients with DLBCL. The first advance was the introduction of anthracycline-containing chemotherapy in the mid-1970s [2]. Combination chemotherapy consisting of cyclophosphamide, doxorubicin, vincristine, and prednisone (CHOP) proves the best available treatment for DLBCL patients in the subsequent clinical trial [3]. The second is the addition of rituximab, the monoclonal anti-CD20 antibody, to the CHOP chemotherapy, which further improves the outcome [4]. At least 50% of DLBCL patients achieved long-term disease-free survival [5].

Despite breakthroughs in treatment, approximately 30–40% of patients with DLBCL undergo relapses eventually [6, 7]. Patients with refractory DLBCL or a relapse within 1 year show dismal outcomes compared to those with a late relapse [8]. According to previous literature, the risk for death doubles for early relapses within 1 year compared to those who relapsed later. Only 13% of patients who relapsed within 1 year were alive after a follow-up of 8 years. Besides, these patients had a lower overall response to salvage regimens [9]. With the advent of CAR-T, the survival of refractory/relapsed DLBCL patients was prolonged significantly compared with standard care, especially those who relapsed within 1 year [10].

Identifying risk factors associated with the very early relapse occurring within 1 year is significant in filtering out potential patients who may benefit from CAR-T therapy. Hitherto, very few series of patients who relapsed within 1 year after complete remission have been published. This study aims to investigate the incidence, clinical features, and overall survival of DLBCL patients with an early relapse in China.

## Patients and methods

### Study population

All consecutive patients with definite diagnoses of DLBCL between March 2009 and April 2017 at our center were included. Patients with incomplete data on treatment response were excluded. Primary central nervous system lymphomas (PCNSL) at diagnosis were excluded for heterogeneity. The diagnosis of DLBCL was confirmed independently by two experienced pathologists based on the World Health Organization classification criteria for hematopoietic and lymphoid tissue tumors [11]. CR was defined according to the 1999 Cheson criteria [12]. The study was approved by the Ethics Committee of Peking Union Medical College Hospital.

### Endpoints and assessment

In our study, the primary endpoint was the occurrence of relapse. Relapse was defined as the recurrence of the disease in patients who achieved CR after the initial treatment. Primarily refractory patients who present progressive disease or stable disease as the best response to chemotherapy were excluded from our study. Relapse was preferably demonstrated by rebiopsy of involved sites. Patients without pathological proof were also considered to relapse if there was evident progression on fluorodeoxyglucose positron-emission tomography (FDG-PET) in concordance with aggressive clinical presentation. Early relapse in our study was defined as relapse within 1 year. The survival endpoint was also assessed. Overall survival (OS) was calculated from the date of diagnosis until the death or date of the last follow-up.

### Data collection

Clinical data of relapse patients were collected including age, gender, cell of origin(COO) based on Hans’ classification, Eastern Cooperative Oncology Group performance score (ECOG PS), Ann Arbor stage, international prognostic index (IPI) score, extranodal involvement, bone marrow involvement, lactate dehydrogenase (LDH), β2-microglobulin (β2-MG), EBV DNA, lymphocyte/monocyte ratio (LMR), initial treatment, and relative dose. The relative dose (RD) was calculated as the mean RD for cyclophosphamide and doxorubicin: RD = (actual dose) / (planned dose).

### Follow-up

Follow-up was conducted via telephone, review of patient records, and the electronic follow-up system at our hematology department for lymphoma patients. All study subjects were followed from the date of lymphoma diagnosis until death or the end of the study period (Oct 2022), whichever occurred first.

### Statistical analysis

Statistics between-group categorical data were compared using the Chi-square test or Fisher’s exact probability test. Factors with* p *< 0.1 in the univariate analysis were selected for inclusion in the multivariate logistic regression analysis. Survival analysis was performed using the Kaplan–Meier method. Data were analyzed using GraphPad 9.0 (GraphPad, San Diego, CA, USA) and SPSS 22.0 statistical software (IBM Corp., Armonk, NY, USA). Differences with *P* < 0.05 were considered statistically significant.

## Results

### Demographic features

**Fig. 1:**
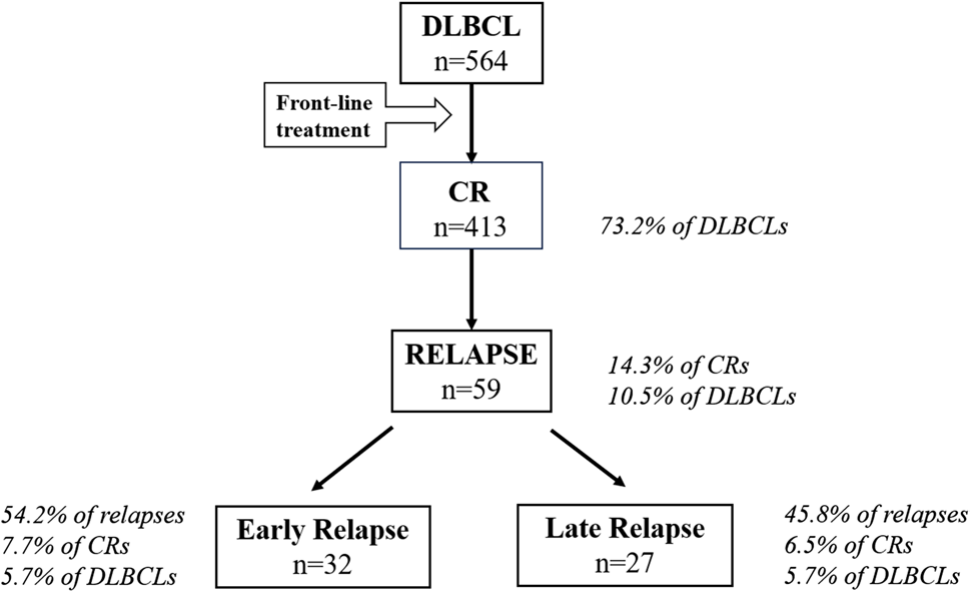
Flowchart of patients relapsing after a first complete remission

We identified 670 patients diagnosed with DLBCL from our center. Patients with incomplete data on treatment response (*n* = 65) and patients diagnosed with PCNSL (*n* = 41) were excluded. Eventually, 564 patients were included. After first-line treatment, 413 patients obtained CR. With a median follow-up of 65.1 months (range: 0.1–159.3 months), 59 patients relapsed: 32 patients (54.2%) relapsed within 1 year, and 27 patients (45.8%) relapsed 1 year or more from CR1(Fig. 1). Patients undergoing an early relapse had a median age of 60 years (range 28–77), while the median age of the patients experiencing relapse after 1 year was 59 years (range 20–81). The male-to-female ratio was 1:1 and 0.6:1, respectively. The difference in age and gender was non-significant between the two groups. Detailed clinical, paraclinical, and treatment features are described in Table 1.

**Table 1:** Characteristics of patients relapsing after a CR achieved with initial treatment

Characteristics	Total	Relapse < 1y	Relapse ≥ 1y	P*-value(Fisher’s exact)
	*n* = 59	*n* = 32	*n* = 27	
*Age, years; median(range)*		60 (28–77)	59 (20–81)	0.797
≤ 60	27 (45.8%)	14 (43.8%)	13 (48.1%)	
> 60	32 (54.2%)	18 (56.2%)	14 (51.9%)	
*Gender*				
Male	26 (44.1%)	16 (50.0%)	10 (37.0%)	0.430
Female	33 (55.9%)	16 (50.0%)	17 (63.0%)	
*Cell of origin*				
GCB	24 (40.7%)	15 (46.9%)	9 (33.3%)	0.555
ABC	24(40.7%)	12 (37.5%)	12 (44.5%)	
Unknown	11 (18.6%)	5 (15.6%)	6 (22.2%)	
*ECOG PS*				
0–1	25 (42.4%)	9 (28.1%)	16 (59.3%)	0.020
≥ 2	34 (57.6%)	23 (71.9%)	11 (30.7%)	
*Ann Arbor stage*				
I–II	6 (10.2%)	1 (3.1%)	5 (18.5%)	0.084
III–IV	53 (89.8%)	31 (96.9%)	22 (81.5%)	
*IPI*				
0–2	25 (42.4%)	8 (25.0%)	17 (63.0%)	0.004
3–5	34 (57.6%)	24 (75.0%)	10 (37.0%)	
*Extranodal involvement*				
0–1	37 (65.0%)	21 (65.6%)	18 (66.7%)	> 0.999
≥ 2	20 (35%)	11 (34.4%)	9 (33.3%)	
*Bone marrow involvement*				
Yes	16 (27.1%)	9 (28.1%)	7 (25.9%)	> 0.999
No	43 (72.9%)	23 (71.9%)	20 (74.1%)	
*LDH*				
Elevated	28 (50.9%)	21 (65.6%)	7 (28%)	0.002
Normal	27 (49.1%)	11 (34.4%)	16 (72%)	
*EBV DNA*		*n* = 18	*n* = 19	0.433
Elevated	3 (8.1%)	2 (11.1%)	1 (5.3%)	
Normal	34 (91.9%)	16 (88.9%)	18 (94.7%)	
LMR;median (IQR)		2.75 (1.28–4.14)	2.45 (1.62–4.22)	0.803
*RD*				
100%	37 (62.7%)	17 (53.1%)	20 (74.1%)	0.114
75–100%	22 (37.3%)	15 (46.9%)	7 (25.9%)	
*First-line ASCT*				
Yes	8 (13.6%)	6 (18.5%)	2 (7.4%)	0.269
No	51 (76.4%)	26 (81.5%)	25 (92.6%)	
*Combined radiotherapy*				
Yes	13 (22.0%)	9 (28.1%)	4 (14.8%)	0.345
No	46 (78%)	23 (71.9%)	23 (85.2%)	

*P-value is calculated using the Chi-square test or Fisher’s exact test, differences with *P* < 0.05 are considered statistically significant

### Risk factors

We conducted a comparison of the clinical characteristics between the two groups. Patients relapsing in 1 year showed an inferior risk profile at diagnosis: elevated LDH level (*P* = 0.002), high ECOG PS (*P* = 0.02), and high IPI (*P* = 0.004). No significant difference was identified in cell of origin, Ann Arbor stage, extranodal involvement, bone marrow involvement, β2-MG, and LMR. EBV DNA was available for 37 cases. However, no distinction was detected between two groups.

Initial treatment was similar in the two groups. CHOP or CHOP-like chemotherapy combined with rituximab was adopted with curative intent. Dosage reduction to a relative dose of 75–100% was made in patients who could not tolerate adequate regimens after geriatric assessments. Reduction in dosage was more common in patients with relapse within 1 year (46.9%). In the other group, 25.9% of patients received a reduced regimen. Overall, 13 patients received adjuvant radiotherapy and 8 patients received front-line autologous stem cell transplant (ASCT) as consolidation. Nevertheless, no statistical difference was found.

Logistic regression analysis was conducted to identify the risk factors for early relapses. Factors with *P* < 0.1 in the univariate analysis were selected for inclusion in the multivariate logistic regression analysis. The results show that a higher ECOG PS and IPI score were closely associated with early relapses (Table 2).

**Table 2:** Risk factors for early relapses after initial treatment in DLBCL patients

Variable	Univariate analysis		Multivariate analysis	
	OR (95% CI)	*p*-value	OR (95% CI)	P*-value
Age	0.838 (0.299–2.343)	0.736		
Gender	1.7 (0.598–4.830)	0.319		
COO	1.234 (0.602–2.528)	0.566		
ECOG PS	0.269 (0.091–0.798)	0.018	0.274 (0.08–0.936)	0.039
Ann Arbor stage	0.293 (0.052–1.654)	0.165		
IPI	0.196 (0.064–0.6)	0.004	0.227 (0.066–0.775)	0.018
Extranodal involvement	0.955 (0.323–2.819)	0.933		
Bone marrow infiltration	1.118 (0.352–3.550)	0.85		
LDH	4.364 (1.382–13.772)	0.012		
EBV DNA	2.25 (0.299–2.352)	0.524		
LMR	0.976 (0.808–1.178)	0.799		
ASCT	0.45 (0.151–1.343)	0.22		
Combined radiotherapy	2.885 (0.531–15.662)	0.152		

*P-value calculated from logistic regression analysis, differences with *P* < 0.05 considered statistically significant

### Treatment at relapse

The relapse was biopsy-proven in 25 of 59 patients (42.4%). All of them relapsed with DLBCL histology. Salvage regimens were diverse and are shown in Table 3. Thirty-eight of 59 patients (64.4%) with relapse chose a rituximab-containing regimen as second-line treatment. Chemotherapies such as DHAP (dexamethasone, cisplatin, and cytarabine), GDP (gemcitabine, dexamethasone, and cisplatin), ICE (ifosfamide, carboplatin, and etoposide), ESHAP (etoposide, methylprednisolone, cytarabine, cisplatin), GemOx (gemcitabine, oxaliplatin), and MINE (mesna, ifosfamide, mitoxantrone, etoposide) were given to patients. Only 4 patients in our study received second-line ASCT afterward. A considerable proportion of patients with relapse lost eligibility for ASCT due to a poor response to classic salvage chemotherapy (*n* = 16) or a rapid decline in clinical status (*n* = 25). Another barrier to accessing ASCT was the relevant high cost (*n* = 10). Programmed cell death protein 1 (PD-1) blockade and lenalidomide were attempted in a small fraction of patients with relapse. Since CAR-T therapy was CFDA-approved in 2021 for the treatment of refractory/relapsed DLBCL, only one patient who relapsed within 1 year received CAR-T therapy.

**Table 3:** Treatment at relapse

Treatment at relapse	relapse < 1y	relapse ≥ 1y
	*n* = 32	*n* = 27
Salvage chemotherapy	*n* = 31	*n* = 22
DHAP	8	5
GDP	4	2
GemOx	1	0
ICE	4	2
ESHAP	2	1
MINE	5	2
Others	7	10
Rituximab	*n* = 32	*n* = 27
Yes	19	19
No	13	8
Lenalidomide	3	4
PD-1 inhibitor	0	1
CAR-T therapy	1	0
ASCT	1	3

### Overall survival

**Fig. 2:**
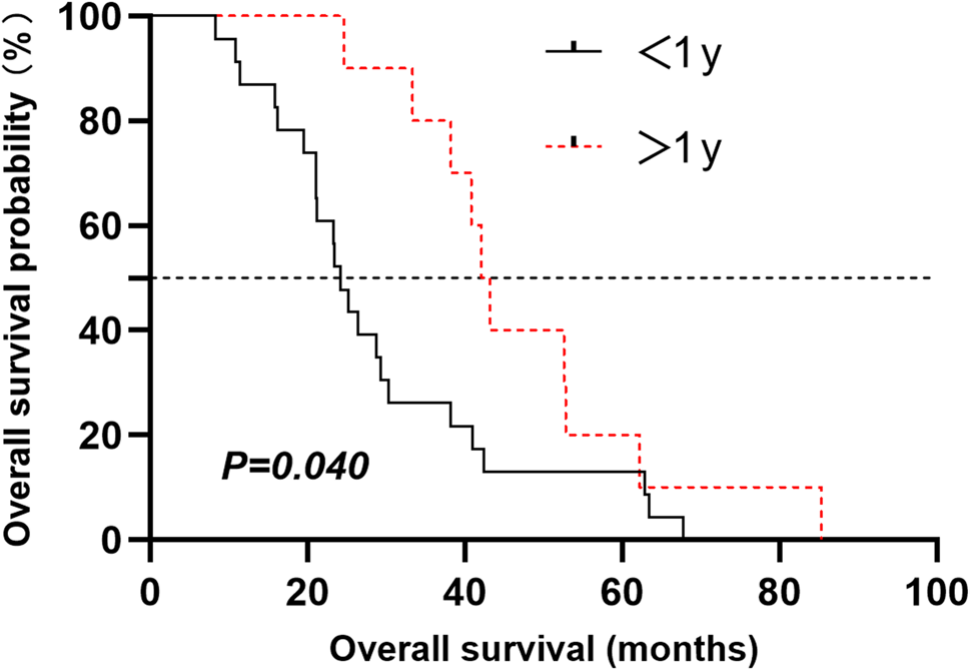
Kaplan–Meier estimates of overall survival from the first relapse following a complete remission after front-line treatment in patients with DLBCL

A trend of better five-year OS was observed in patients who relapsed after 1 year than those who relapsed within 1 year (median OS, 42.65, and 24.2 months, respectively; log-rank test, *P* = 0.04; Fig. 2). Multivariate analysis for OS showed that relapse within 1 year was an independent parameter for reduced overall survival (HR 0.241, 95% CI 0.1–0.583, *P* = 0.002, Table 4). Both ECOG PS and IPI scores failed to be statistically significant in COX regression analysis though they are significant in univariate analysis. We failed to demonstrate a beneficial effect of ASCT on the outcome since only 4 patients (6.7%) received second-line ASCT after salvage chemotherapy. Noticeably, the only patient who adopted CAR-T therapy achieved a second CR and was alive till the last follow-up, indicating the remarkable efficacy of CAR-T therapy on relapsed, especially, early elapsed DLBCL patients.

**Table 4:** Prognostic factors for patients with DLBCL

Variable	Univariate analysis	Multivariate analysis	
	*P*-value	HR(95% CI)	P*-value
Age	0.838		
Gender	0.107		
COO	0.26		
ECOG PS	0.094	1.253 (0.572–2.741)	0.573
Ann Arbor stage	0.19		
IPI	0.044	0.957 (0.392–2.333)	0.922
Extranodal involvement	0.949		
Bone marrow infiltration	0.2211		
LDH	0.354		
EBV DNA	0.778		
Combined radiotherapy	0.706		
ASCT	0.283		
Relapse	0.002	0.241 (0.100–0.583)	0.002

*P-value calculated from COX regression analysis, differences with *P* < 0.05 considered statistically significant

## Discussion

According to previous studies, patients with DLBCL usually relapse early, within 1–2 years from diagnosis [6, 13]. Patients relapsing within 1 year from remission have a poor prognosis resembling refractory patients and are less likely to respond to second-line chemotherapy compared to those relapsing beyond 1 year [8, 9]. The emerging CAR-T therapy shows promise in the front line for these patients and is recommended as the preferred choice for patients who relapsed within 1 year in the latest National Comprehensive Cancer Network guidelines. Besides, CAR-T therapy is demonstrated to be highly effective as part of first-line therapy for high-risk DLBCL, with a manageable safety profile [14, 15]. So it is important to distinguish patients prone to relapse early. Few studies address this issue in the Chinese population in the post-rituximab era.

In our study, patients relapsing within 1 year show different clinical characteristics from those with late relapses at diagnosis. They enjoyed a higher IPI score and worse performance scores. Elevation of LDH was seen more often in them. Other baseline features such as extranodal or bone marrow involvement, EBV DNA, β2-MG, and LMR are also included for analysis. However, no significant difference was demonstrated. All patients received standard R-CHOP therapy. For the elderly intolerant to adequate therapy, a minor reduction was made to a relative dosage of 75–100% with curative intent. We observed a higher percentage of reduced chemotherapy in the early relapse group (46.9% vs. 25.9%) though no significant difference was found between the two groups. In a previous study, radiotherapy was identified to lower the occurrence of relapses within 2 years from diagnosis [16]. However, we failed to verify its effect on the time of relapse in our study, which may be attributed to the limited sample size. Front-line ASCT seems to have a limited protective effect on late relapse. In a multivariate logistic regression analysis, we found that a higher ECOG PS and IPI score were closely associated with early relapses.

As expected, patients relapsing within 1 year have a worse OS than those with late relapse. Moreover, we found that time to relapse was an independent prognostic factor for overall survival, in accordance with the previous study [9]. With regard to second-line therapy, the majority of patients in our study received salvage chemotherapy according to standard care [8, 17]. For those who were not fit for chemotherapy, we provided chemo-free approaches with favorable toxicity profiles. PD-1 inhibitor and lenalidomide were attempted. Secondary ASCT was preferred when a secondary response was achieved for eligible patients. Only one person in our cohort was administered CAR-T therapy. This patient achieved CR and was still alive till the last follow-up, expanding curative options for this population.

In conclusion, patients relapsing within 1 year enjoyed an inferior risk profile at diagnosis: elevated lactate dehydrogenase level, high ECOG score, and high IPI score. In the multivariate logistic regression analysis, ECOG score, and high IPI score are associated with early relapse. Our analysis has shown that the prognosis of early relapsing patients is worse than that of those relapsing later. Time to relapse is an independent prognostic factor for patients with DLBCL. Genetic landscapes associated with early relapse patterns remain to be explored further.
